# Beyond the walls of the classroom: the psychodynamics of professional commitment and job involvement among female primary school teachers

**DOI:** 10.3389/fpsyg.2024.1465880

**Published:** 2024-10-18

**Authors:** Qiaolan Su

**Affiliations:** School of Tourism and Sports Health, Hezhou University, Hezhou, China

**Keywords:** professional commitment, self-efficacy, psychological resilience, perceived organizational support, job involvement, female teachers

## Abstract

This research used structural equation modeling (SEM) to explore the relationship between professional commitment, self-efficacy, psychological resilience, perceived organizational support and job involvement of female teachers in Chinese primary schools. The purpose of the study was to understand how these psychological and organizational factors work together on teachers’ job involvement. Based on the questionnaire survey of 596 female teachers in primary schools, the data were collected and analyzed by AMOS software. The results show that professional commitment plays a central role in teachers’ job involvement, and self-efficacy and psychological resilience, as important psychological resources, significantly affect job involvement through the mediation of perceived organizational support. The model fitting indicators CFI, RMSEA and SRMR were in line with the acceptance criteria, which verified the adaptability of the hypothesis model. The findings not only reinforce the application of resource conservation theory in the field of education, but also provide school administrators with strategies to improve teachers’ job involvement, especially by enhancing the importance of organizational support and resilience. In addition, the results have practical implications for the design of targeted teacher professional development programs.

## 1 Introduction

Female primary school teachers play a crucial role in shaping the educational landscape in China. Recent statistics from the Ministry of Education indicate that female teachers account for approximately 60% of the primary school workforce in urban areas and 70% in rural regions (Ministry of Education, 2023). Job involvement is a critical factor in determining the effectiveness of teaching and learning processes (Atteh et al., 2020). Teachers who are highly involved in their jobs not only perform better but also positively influence student outcomes and the overall school environment (Liu and Yu, 2024; Wang, 2024). In particular, the job involvement of female primary school teachers has been a growing focus of research, as these educators often face additional challenges related to work-life balance and societal expectations (Skaalvik and Skaalvik, 2014; Wang, 2024). Recent studies have demonstrated that job involvement is closely linked to self-efficacy, resilience, and perceived organizational support, all of which can mitigate the stress associated with teaching and improve overall professional commitment (Turner et al., 2022). These psychological and organizational resources are critical for enhancing teacher well-being and engagement, especially in high-stress environments such as primary education (Giancaspro et al., 2022; Wang, 2024).

Research on job involvement has traditionally focused on Western educational contexts, where cultural expectations and gender roles differ significantly from those in non-Western settings (Wang and Hall, 2018). Kumar et al. (2024) highlighted that organizational culture and leadership have a profound impact on organizational outcomes, and this relationship is often mediated by the cultural context in which the organization operates. For example, Wang (2024) notes that in China, female teachers often face added pressures from traditional gender expectations, which may negatively influence their professional commitment and job involvement. Studies like Tschannen-Moran and Hoy (2001) have shown that teachers with higher self-efficacy tend to demonstrate greater resilience and engagement in their work, but these studies often fail to account for how cultural factors influence the dynamics of teacher engagement in non-Western contexts.

Furthermore, while the role of perceived organizational support in reducing burnout has been well-documented, its role as a mediator between professional commitment and job involvement has been less explored in educational settings, particularly in China (Wang and Pan, 2023). Kanaan et al. (2024) further underline the importance of organizational factors in improving performance, particularly in entrepreneurial firms, by demonstrating that knowledge management and stakeholder relationships play a critical role in moderating the effects of open innovation on firm performance. Saleem et al. (2023) demonstrated that resilience plays a crucial role in maintaining employee performance during challenging situations like the COVID-19 pandemic, suggesting that perceived organizational support could similarly buffer the effects of stress on teacher engagement in high-pressure environments. Liu and Yu (2024) identified perceived organizational support as a key factor in enhancing resilience, suggesting that supportive leadership can foster greater job involvement. Similarly, Edinger and Edinger (2018) emphasized the importance of institutional support in improving job satisfaction and reducing teacher turnover intentions, underscoring the need for further research into how these dynamics unfold in diverse educational contexts. These findings echo the results of both Allam et al. (2021) and Allam et al. (2022), who suggest that the organizational climate, including spiritual and emotional support, plays a critical role in moderating stress and burnout in high-demand work environments like teaching.

In line with the Job Demands-Resources (JD-R) Model, the interplay between job demands and available resources significantly influences job involvement. Bakker and Demerouti (2008) argue that job resources such as organizational support and professional development opportunities can mitigate the negative impact of job demands on teacher engagement. However, the applicability of the JD-R model to non-Western educational settings remains underexplored. The findings of Wang (2024) support the extension of this model to non-Western settings, particularly in understanding how organizational resources enhance resilience and self-efficacy in female primary school teachers in China. Additionally, the Conservation of Resources (COR) Theory posits that individuals strive to retain, protect, and build resources, which can help buffer against job stress and burnout (Hobfoll et al., 2018). This theory has been used to explain how teachers use internal resources like self-efficacy and external resources such as organizational support to enhance their job involvement. The work of Wang and Pan (2023) extends COR theory by exploring how job crafting and organizational resources interact to shape professional commitment, suggesting that self-efficacy and resilience serve as key personal resources for teachers to navigate the challenges of the profession.

Despite the significant role of job involvement in teacher performance, the literature lacks comprehensive studies examining how these psychological and organizational factors interact to influence job involvement, particularly in non-Western educational settings such as China. Most existing research has focused on Western contexts, leaving a gap in understanding how cultural expectations and gender roles affect job involvement among female teachers in China (Liu and Yu, 2024). Additionally, while studies have identified the importance of self-efficacy and resilience, the mediating role of perceived organizational support in this relationship remains underexplored.

### 1.1 Research gaps

The present study addresses several important research gaps. First, it examines the unique socio-cultural challenges faced by female primary school teachers in China, where traditional gender expectations may influence their job involvement and professional commitment. Second, while previous studies have demonstrated the impact of self-efficacy and resilience on job involvement, there is a lack of research on how these factors interact with organizational support in enhancing teacher engagement, particularly among female educators. Finally, this study aims to fill the gap in understanding how perceived organizational support serves as a mediating mechanism between professional commitment and job involvement.

### 1.2 Research questions

How does professional commitment influence job involvement among female primary school teachers in China?

What role does perceived organizational support play in mediating the relationship between professional commitment and job involvement?

How do self-efficacy and resilience contribute to job involvement, and how do they interact with perceived organizational support?

### 1.3 Research objectives

To investigate the impact of professional commitment on job involvement.

To explore the mediating role of perceived organizational support in the relationship between professional commitment and job involvement.

To analyze the contributions of self-efficacy and resilience in enhancing job involvement and their interaction with perceived organizational support.

By addressing these gaps, this study will contribute to a deeper understanding of how psychological and organizational factors influence the job involvement of female primary school teachers in non-Western settings, offering valuable insights for policymakers and school administrators aiming to enhance teacher engagement and well-being.

## 2 Theoretical deduction and research hypothesis

### 2.1 Theoretical deduction

Resource Conservation Theory (COR) provides a powerful framework for analyzing how individuals manage and conserve the resources that are of value to them in order to cope with stress and improve performance (Hobfoll et al., 2018). In the field of education, especially for female teachers in primary schools, they face many challenges in balancing the demands of their profession with the demands of their personal lives. These challenges not only consume their psychological and emotional resources, but also may affect their professional commitment and job involvement.

The career of female primary school teachers is often influenced by multiple pressures, including teaching load, expectations of students and parents, and responsibilities in personal life (Skaalvik and Skaalvik, 2011). When these teachers perceive an imbalance between career engagement and support they receive, they may experience a sense of loss of resources, leading to burnout and reduced job efficacy. In addition, if the work environment does not provide sufficient support, such as low perceived organizational support or limited career development opportunities, teachers’ professional commitment may be affected, thereby affecting their job involvement (Liu et al., 2021; Sun et al., 2022).

In this context, teachers’ self-efficacy and psychological resilience become key internal resources that help them maintain professional commitment and effectively manage conflicts between their work and personal lives. Teachers with strong self-efficacy are more confident to cope with challenges in teaching, while psychological resilience enables them to maintain resilience and positive attitudes in the face of stress (Bandura and Wessels, 1997; Luthans et al., 2010). Studies have shown that when teachers have high self-efficacy and psychological resilience, they are more effective in using personal and professional resources to maintain or even enhance job involvement (Tian et al., 2019).

Therefore, through in-depth understanding and supporting the resource management strategies of female teachers in primary schools, we can help them better cope with the needs of their professional and personal lives, thereby improving their job satisfaction and teaching effectiveness. The purpose of this study was to explore how professional commitment, perceived organizational support, self-efficacy, and psychological resilience collectively affect the job involvement of female primary school teachers, with a view to providing theoretical and empirical support for educational administration and policy making.

### 2.2 Research hypothesis

#### 2.2.1 The relationship between professional commitment and job involvement

According to resource conservation theory, individuals tend to protect, build, and accumulate resources that are valuable to them (Hobfoll et al., 2018). In the field of education, teachers’ professional commitment itself can be regarded as an important psychological resource, which not only affects teachers’ personal professional identity and satisfaction, but also is the core driving force of their daily work motivation. When teachers have a higher commitment to their profession, they are more likely to devote their time, energy and even emotions to their work, thus improving the level of job involvement. This commitment is not only reflected in the quality of teaching activities, but also in the interaction with students and parents and educational innovation (Klassen and Chiu, 2010). Recent studies have also shown that teachers with high professional commitment show higher resilience and positive coping strategies in the face of educational challenges, which further promotes their continuous investment and development in their career (Liu et al., 2021). Therefore, this study hypothesized that professional commitment could significantly and positively affect the level of teachers’ job involvement by enhancing their sense of value and satisfaction in the educational profession.

*H1*: Occupational commitment has a significant positive impact on job engagement.

#### 2.2.2 The relationship between professional commitment and perceived organizational support

According to the theory of resource conservation, individuals see the positive outlook of their personal value and career in positive professional attitudes and commitments, so they are more inclined to seek and use external support that can help them protect and increase these resources, such as organizational support (Hobfoll et al., 2018). Teachers with high professional commitment are more likely to feel and rely on various supports provided by schools and educational institutions, such as training, resources and emotional support, because of their deep investment in education, which not only improves their work efficiency, but also enhances their job satisfaction and sense of belonging (Greenberg et al., 2020). In addition, when teachers’ professional commitment is recognized, organizations usually respond to this commitment by providing more resources and support, thus forming a positive feedback loop and enhancing teachers’ sense of organizational support (Buhari et al., 2020). This dynamic relationship suggests that professional commitment can be used as a psychological resource, and its enhancement can directly enhance teachers’ perceived organizational support, which, in turn, further consolidates and enhances teachers’ professional commitment. Therefore, this study hypothesized that there was a positive relationship between professional commitment and perceived organizational support among female primary school teachers, that is, the higher the professional commitment, the stronger the perceived organizational support.

*H2*: Professional commitment has a significant positive effect on perceived organizational support.

#### 2.2.3 Relationship between career commitment and resilience

According to the theory of resource preservation (Hobfoll et al., 2018), individuals tend to actively maintain and increase psychological resources that contribute to their personal and professional development. In the field of education, psychological resilience is an important psychological resource for teachers to face the challenges and pressures of daily teaching. Teachers’ professional commitment reflects their love and devotion to the profession of education, which can enhance their psychological resilience, because high commitment makes teachers more motivated to overcome difficulties and challenges in their profession (Luthans et al., 2010). Research has shown that teachers with high commitment are more resilient and adaptable when confronted with teaching or management challenges, because they see these challenges as opportunities to achieve their career goals rather than insurmountable obstacles (Klassen and Chiu, 2010). This kind of mental resilience not only helps them maintain their professional commitment, but also promotes them to seek innovation and improvement in educational practice, thereby improving the quality of education and student satisfaction. In addition, the enhancement of psychological resilience is also positively related to teachers’ personal accomplishment and career satisfaction, which are important factors of professional commitment (Kim et al., 2021). Therefore, this study proposes that the professional commitment of female teachers in primary schools helps them to cope with the stress and challenges in the educational process more effectively by enhancing psychological resilience, which is expected to be verified in empirical research. An in-depth exploration of this relationship helps to understand how teachers can optimize the durability and success of their careers by improving mental resilience.

*H3*: Professional commitment has a significant positive effect on psychological resilience.

#### 2.2.4 The relationship between career commitment and self-efficacy

According to resource conservation theory, individuals tend to protect and accumulate resources that are valuable to them in order to reduce the loss of resources and enhance their likelihood of survival and success (Hobfoll et al., 2018). Teachers’ self-efficacy is a key psychological resource in educational settings that describes teachers’ beliefs about their ability to accomplish teaching tasks and overcome teaching challenges. This belief is particularly important in teachers’ daily teaching activities, because it directly affects teachers’ teaching behavior and students’ learning outcomes (Bandura and Wessels, 1997). Professional commitment, as a deep emotion and identity, can significantly enhance teachers’ self-efficacy. When teachers have a strong commitment to their profession, they are more inclined to devote themselves to teaching practice, which not only increases their experience, but also improves their ability to deal with complex situations, thus enhancing their self-efficacy (Skaalvik and Skaalvik, 2014). In addition, teachers with high professional commitment are generally more willing to engage in ongoing professional development activities that further enhance their teaching skills and self-confidence, thereby reinforcing self-efficacy (Bandura and Wessels, 1997). Recent research also supports this view, showing that there is a significant positive link between teachers’ professional commitment and self-efficacy, and this link is particularly important in the face of educational reform and student diversity challenges (Lestari et al., 2021). Therefore, this study anticipates that the promotion of professional commitment will significantly enhance the self-efficacy of female teachers in primary schools, which will help them participate in teaching activities more effectively and improve the quality of teaching.

*H4*: Professional commitment has a significant positive effect on self-efficacy.

#### 2.2.5 Relationship between perceived organizational support and job involvement

According to resource preservation theory, individuals strive to acquire and protect resources that help reduce stress, prevent resource loss, and promote individual well-being (Hobfoll et al., 2018). Organizational support is an important external resource in the educational environment, which includes the provision of necessary physical resources, emotional support, career development opportunities, and effective school management and leadership. These supports can help teachers perform their teaching duties more effectively and reduce occupational stress, thereby enhancing their job involvement (Giancaspro et al., 2022). Specifically, when teachers perceive a high level of organizational support, such as through professional development training, timely feedback and recognition, and adequate teaching resources, they are more likely to feel empowered and supported, which directly enhances their professional self-esteem and satisfaction. This feeling not only increases the professional commitment of teachers, but also stimulates greater enthusiasm and participation in their work (Sun et al., 2022). In addition, organizational support can also alleviate the conflict between work and personal life, and positively affect job involvement by improving job satisfaction and quality of life (Ratnasari, 2021). Therefore, this study hypothesized that the perceived support provided by organizations to female primary school teachers would significantly increase their job involvement. This commitment is reflected not only in daily teaching activities, but also in the long-term commitment of teachers to school development and student growth. The exploration of this relationship will provide school administrators with strategies on how to enhance teachers’ job involvement by enhancing organizational support.

*H5*: Perceived organizational support has a significant positive impact on job involvement.

#### 2.2.6 Relationship between psychological resilience and job involvement

From the perspective of resource conservation theory, psychological resilience can be viewed as a personal resource that enables individuals to prevent the loss of resources in the face of occupational stress and challenges and to cope effectively with adversity (Hobfoll et al., 2018). Psychological resilience not only helps teachers to cope with the daily pressure of teaching, but also promotes their persistence and creativity in work, thus improving job involvement (Luthans et al., 2010). Specifically, teachers with high mental resilience were able to recover more quickly from setbacks and difficulties, and they tended to view challenges as opportunities for growth and learning. This positive attitude not only enhances their job satisfaction, but also stimulates their initiative and innovation in educational practice (Skaalvik and Skaalvik, 2011). Studies have shown that teachers with high resilience are more able to maintain their sense of efficiency and motivation in teaching activities, which directly affects their teaching quality and student learning outcomes (Lestari et al., 2021). Therefore, this study anticipates that psychological resilience will have a significant positive impact on job involvement among primary school teachers. By enhancing psychological resilience, teachers can not only better cope with the pressure and challenges in their work, but also actively participate in educational innovation and school development, thus improving the overall quality and efficiency of education. The validation of this hypothesis will facilitate the development of resilience training and support procedures for teachers to enhance their career satisfaction and job performance.

*H6*: psychological resilience has a significant positive effect on job involvement.

#### 2.2.7 The relationship between self-efficacy and job involvement

According to the theory of resource conservation, self-efficacy is an important personal resource, which involves individuals’ beliefs about their ability to accomplish specific tasks (Hobfoll et al., 2018). This belief is a key factor in teachers’ ability to maintain a high level of engagement and positive performance in the face of educational challenges. Teachers with high self-efficacy believe that they can effectively manage the classroom and stimulate student learning, and this belief motivates them to adopt more active strategies in the teaching process, thereby increasing their job involvement and satisfaction (Bandura and Wessels, 1997). Research has shown that teachers with a strong sense of self-efficacy are more likely to engage in teaching activities that require a high degree of cognitive and emotional involvement because they feel more capable of facing and overcoming challenges in these activities (Tian et al., 2019). In addition, this self-efficacy is also associated with lower burnout and higher job satisfaction, which further demonstrates its positive impact on job involvement (Wang and Hall, 2018). Self-efficacy not only enhances teachers’ confidence in their professional ability, but also elevates their expectations of teaching results, so as to maintain persistence and innovation in the face of students’ learning problems and teaching challenges. Therefore, this study predicts that the self-efficacy of female teachers in primary schools will significantly and positively affect their job involvement. By improving teachers’ self-efficacy, we can effectively increase their teaching activity and educational achievements, thus improving the overall quality of teaching and the educational effect of the school.

*H7*: Self-efficacy has a significant positive effect on job involvement.

**Figure 1:**
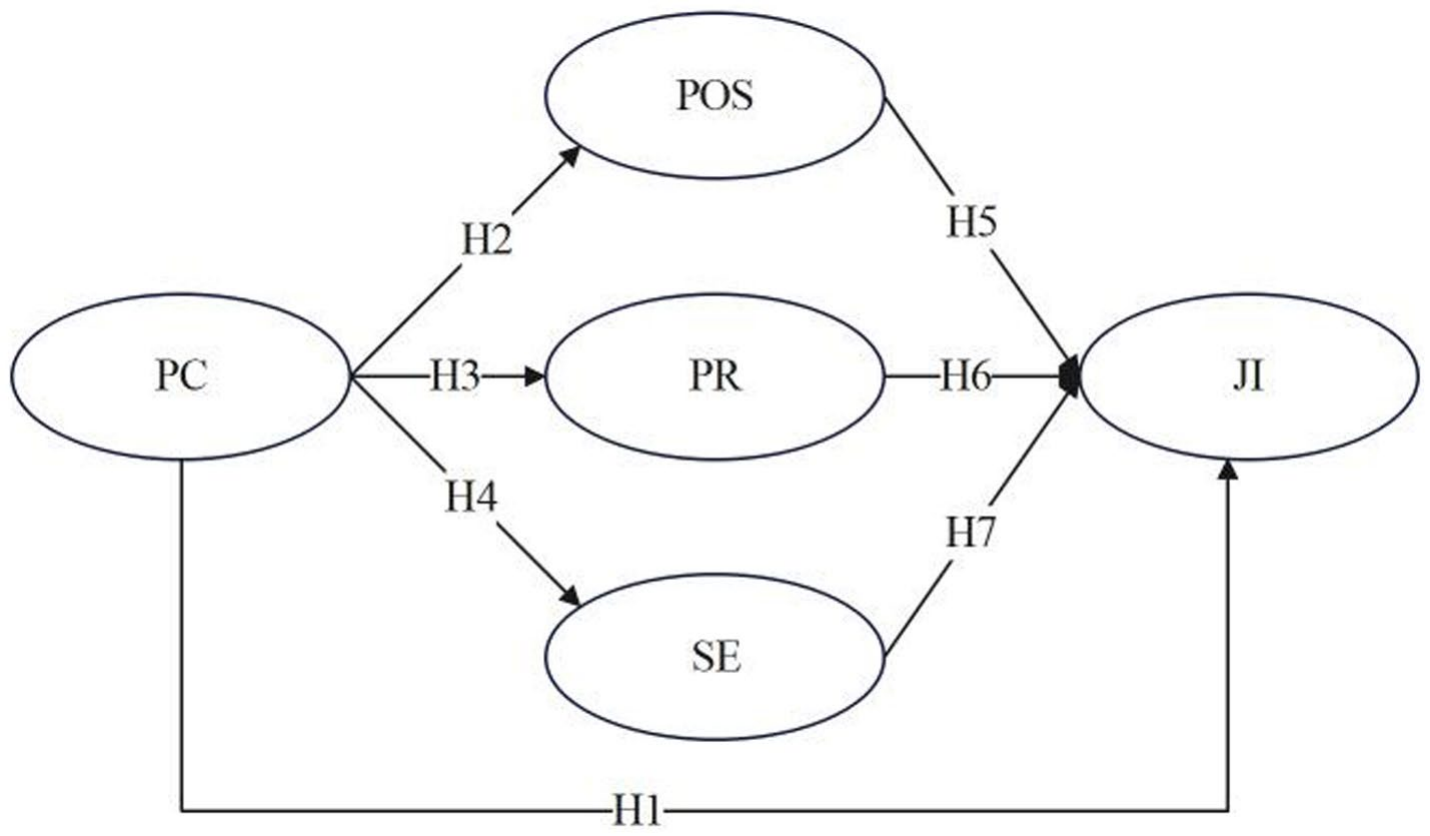
Conceptual model. PC, professional commitment; POS, perceived organizational support; PR, psychological resilience; SE, self-efficacy; JI, job involvement.

Based on the above assumptions, this study constructs a model of the relationship among professional commitment, perceived organizational support, psychological resilience, self-efficacy and job involvement of female primary school teachers (Figure 1).

## 3 Research design

### 3.1 Questionnaire design

The questionnaire includes two modules: the main content and demographic variables. The main content includes five dimensions: professional commitment, perceived organizational support, psychological resilience, self-efficacy and job involvement. The Likert 5-scale was used to measure each dimension. Demographic variables mainly include age, marital status, education level, working experience, professional title and so on.

### 3.2 Measurement of variables

#### 3.2.1 Professional commitment scale

In this study, we adopted the Professional Commitment Scale developed by Suddaby et al. (2009) to assess the degree of professional commitment of female primary school teachers (Suddaby et al., 2009). The scale contains seven items related to the individual’s sense of identity and strength of commitment to their profession. Participants rated each item on a five-point Likert scale (1 = strongly disagree, 5 = strongly agree) to reflect their level of commitment to their current career.

#### 3.2.2 Perceived organizational support scale

Perceived organizational support was assessed using the One-dimensional Perceived Organizational Support Scale (Survey of Perceived Organizational Support, SPOS) developed by Eisenberger et al. (1997). This study chose to use a version of 8 items from the scale, which were streamlined and adjusted to fit this study of female elementary school teachers. Participants rated their perceived organizational support on a five-point Likert scale (1 = strongly disagree, 5 = strongly agree). The scale is widely applicable and authoritative in the current educational environment.

#### 3.2.3 Psychological resilience scale

Psychological Resilience was assessed using the Resilience Scale (CD-RISC-10) developed by Connor and Davidson (2003), a 10-item scale that assesses an individual’s resilience and adaptability in the face of adversity. The scale uses a five-point scale (1 = never, 5 = always), and a high score indicates that an individual has a high level of psychological resilience. The scale has been widely used in a variety of populations and has shown good reliability and validity.

#### 3.2.4 Self-efficacy scale

Self-efficacy was measured according to the teacher self-efficacy scale developed by Tschannen-Moran and Hoy (2001), which covered 12 items related to teachers’ self-confidence in classroom management, motivation of students and cooperation with parents. A five-point Likert scale (1 = none, 5 = very many) was used for scoring. This scale can effectively measure the confidence level of teachers in dealing with daily teaching challenges.

#### 3.2.5 Job involvement scale

Job Involvement was measured by Schaufeli and Bakker’s Simplified Job Involvement Scale (Schaufeli et al., 2006), which has nine items and covers three dimensions of vitality, dedication and focus. Participants were rated on a five-point Likert scale (1 = never, 5 = always). This rating system helps to provide a detailed understanding of the involvement of female primary school teachers in their work.

### 3.3 Data collection and sampling

#### 3.3.1 Sampling method

This study employed a convenience sampling method, selecting female primary school teachers in Hezhou City, Guangxi Zhuang Autonomous Region, as the research subjects through the researcher’s collaboration with the local education system. The use of convenience sampling was based on the limitations of time and resources, while also taking into account the cooperative relationship with the educational authorities, ensuring that a large and valid sample could be obtained quickly (Emerson, 2015). Although convenience sampling offers a practical approach, its representativeness is limited, as the sample may skew toward teachers who are more willing or available to participate, potentially introducing selection bias (Stratton, 2021). Therefore, caution should be exercised when generalizing the results of this study. Future research can enhance representativeness by expanding the sample sources.

#### 3.3.2 Data collection process

The data collection process took place between March 1, 2024, and May 1, 2024, lasting 2 months. Data were gathered from 16 primary schools in Hezhou City, covering both urban and suburban schools to ensure that the data reflected the professional commitment and job involvement of female primary school teachers in various teaching environments. The selection of specific schools was based on two criteria: (1) the willingness of the schools to collaborate with the researcher, and (2) the presence of a sufficient number of female teachers to provide a stable sample size.

During the questionnaire distribution process, the researcher employed both online and offline methods. For online distribution, the researcher used school administrative systems and WeChat groups, sending the questionnaire link to school administrative staff, who then forwarded the link to all female teachers. To ensure wide coverage, the researcher also established communication channels through WeChat and QQ to answer any questions the teachers had while completing the questionnaire.

For offline distribution, paper questionnaires were provided for teachers who could not participate online. The researcher personally visited the schools to distribute paper questionnaires and organized on-site completion during faculty meetings at some schools. All participants were informed that their participation was entirely voluntary and anonymous, with the data being used solely for academic purposes, and strict confidentiality measures were followed throughout the process.

#### 3.3.3 Date overview

A total of 600 questionnaires were collected, of which 596 were valid, with an effective rate of 99.33%. Teachers participating in the survey are representative in terms of age, marital status, educational background, work experience and professional title, which helps to fully understand the current situation of female teachers’ professional commitment and job involvement in primary schools. In terms of age distribution, there are 130 teachers aged 18–29, accounting for 21.81%, 185 teachers aged 30–39, accounting for 31.04%, 190 teachers aged 40–49, accounting for 31.88%, and 91 teachers aged 50 and above, accounting for 15.27%. This age distribution is helpful to analyze the impact of different life cycle stages on career commitment and work engagement. In terms of marital status, there were 108 unmarried teachers, accounting for 18.12%, 467 married teachers, accounting for 78.36%, and 21 divorced teachers, accounting for 3.52%. Marital status may affect teachers’ job involvement and professional commitment, especially married teachers may face the dual pressures of family and work. In terms of education level, there are 4 teachers with senior high school education background or below, accounting for 0.67%; 113 teachers with junior college education background, accounting for 18.96%; 474 teachers with bachelor’s degree, accounting for 79.53%; 5 teachers with master’s degree or above, accounting for 0.84%. This reflects the current prevalence of higher education levels among primary school teachers. In terms of working experience, there are 151 teachers with working experience of 1–5 years, accounting for 25.34%; 140 teachers with working experience of 6–10 years, accounting for 23.49%; 70 teachers with working experience of 11–20 years, accounting for 11.74%; There are 235 teachers with more than 20 years, accounting for 39.43%. This indicates that a considerable number of teachers participating in the survey are experienced senior teachers. The structure of professional titles shows that there are 207 teachers with junior professional titles, accounting for 34.73%; 179 teachers with intermediate professional titles, accounting for 30.03%; 159 teachers with senior professional titles, accounting for 26.68%; There are 51 ungraded teachers, accounting for 8.56%. The structure of professional titles reflects the hierarchy of teachers’ professional development and the difference of their professional achievements. These data provide rich background information for this study, which is helpful to deeply analyze the psychodynamics of professional commitment and job involvement of female teachers in primary schools, and further understand the complex factors behind them and their interactions.

## 4 Data analysis

### 4.1 Reliability and validity of the scale

Confirmatory factor analysis was used to test the reliability and validity of the scale. The reliability was tested by combined reliability (C. R.) and Cronbach’s *α* coefficient. The C. R and Cronbach’s *α* coefficient of each dimension were greater than 0.7, indicating that the internal consistency reliability of the scale was good (Guilford, 1954; Hair et al., 2020). Convergent validity was tested by factor loading and average variance extraction (AVE), and the standardized loading coefficients were all greater than 0.6, and all index factor loadings in the model were significantly different at 0.01, and AVE were all greater than 0.5, indicating that the convergent validity of each dimension of the measurement model was good (Table 1). Discriminant validity means that there is a low correlation or significant difference between the latent trait represented by a latent variable and the traits represented by other latent variables (Hair et al., 2020). The square root of AVE of each latent variable was greater than the correlation coefficient between the latent variable and other latent variables, and the discriminant validity was good (Table 2).

**Table 1:** Reliability and validity of the scale.

Factor	Measured item	Coef.	Std. error	*z* (CR value)	Std. estimate	Reliability	Combined reliability	Convergent validity
PC	PC1	1.000	–	–	0.778	0.931	0.953	0.672
	PC2	1.341	0.061	22.143	0.823			
	PC3	1.419	0.059	24.173	0.881			
	PC4	1.425	0.068	21.048	0.791			
	PC5	1.107	0.048	22.844	0.844			
	PC6	1.162	0.052	22.456	0.833			
	PC7	1.022	0.049	20.753	0.782			
POS	POS1	1.000	–	–	0.906	0.963	0.963	0.767
	POS2	1.027	0.027	38.231	0.924			
	POS3	1.028	0.027	37.777	0.920			
	POS4	0.934	0.026	35.376	0.898			
	POS5	0.829	0.029	28.447	0.819			
	POS6	0.852	0.035	24.661	0.761			
	POS7	0.926	0.027	34.089	0.886			
	POS8	0.884	0.026	33.372	0.878			
PR	PR1	1.000	–	–	0.776	0.965	0.965	0.735
	PR2	1.268	0.052	24.196	0.870			
	PR3	1.167	0.050	23.407	0.849			
	PR4	1.154	0.049	23.763	0.859			
	PR5	1.242	0.054	23.169	0.842			
	PR6	1.307	0.056	23.422	0.849			
	PR7	1.362	0.054	25.185	0.897			
	PR8	1.422	0.058	24.571	0.881			
	PR9	1.310	0.056	23.506	0.852			
	PR10	1.311	0.052	25.017	0.892			
SE	SE1	1.000	–	–	0.704	0.963	0.964	0.689
	SE2	1.141	0.056	20.486	0.860			
	SE3	1.201	0.058	20.790	0.873			
	SE4	1.202	0.058	20.610	0.865			
	SE5	1.213	0.064	19.037	0.798			
	SE6	1.161	0.060	19.484	0.817			
	SE7	1.231	0.063	19.600	0.822			
	SE8	1.112	0.055	20.104	0.844			
	SE9	1.068	0.055	19.461	0.816			
	SE10	1.168	0.059	19.881	0.834			
	SE11	1.191	0.058	20.439	0.858			
	SE12	1.143	0.056	20.417	0.857			
JI	JI1	1.000	–	–	0.88	0.955	0.961	0.734
	JI2	1.039	0.029	35.269	0.924			
	JI3	1.069	0.029	36.679	0.939			
	JI4	1.113	0.031	36.130	0.933			
	JI	1.153	0.040	28.672	0.842			
	JI5	1.174	0.042	27.740	0.828			
	JI6	1.142	0.036	31.617	0.882			
	JI7	1.092	0.036	30.411	0.867			
	JI8	0.863	0.058	14.774	0.545			

**Table 2:** Latent variable discriminant validity test.

	PC	POS	PR	SE	JI
PC	**0.811**				
POS	0.692	**0.876**			
PR	0.490	0.516	**0.866**		
SE	0.434	0.424	0.609	**0.847**	
JI	0.551	0.573	0.717	0.607	**0.847**

The bold diagonal values represent the square root of AVE, and the lower triangle shows the Pearson correlations between dimensions.

### 4.2 Model fitting test

The goodness of fit of the model reflects how well the theoretical model aligns with the actual data (Byrne, 2013; Guilford, 1954; Nunally and Bernstein, 1978). To further improve the model’s fit, we applied the modification indices method by correlating the error terms of items with high residual values (Baumgartner and Homburg, 1996). This technique helps to increase the GFI value while maintaining the theoretical integrity of the model. The test results showed in Table 3. The *χ*^2^/DF value, which indicates a good fit when between 1 and 3, is 3.469, suggesting the model fits the data reasonably well. SRMR (0.04) and RMSEA (0.064) are within the acceptable limits, indicating that the model is adequately specified. For GFI, AGFI, IFI, CFI, and TLI, values close to 1 are ideal, with 0.90 being a typical threshold. The GFI of 0.841 is slightly below the ideal threshold of 0.90. However, it is within the acceptable range as suggested by Doll et al. (1994) and Baumgartner and Homburg (1996), who state that a GFI value above 0.80 can be considered acceptable. Other indices, such as AGFI (0.817), IFI (0.941), CFI (0.941), and TLI (0.936), indicate a good fit of the data to the model. Furthermore, Harman’s single factor exhibited 21.69% shared variance, presenting that the CMV (common method variance) is statistically insignificant (Harman, 1976).

**Table 3:** Model fit test.

Model fitting index	*χ*^2^/df	SRMR	RMSEA	GFI	AGFI	IFI	CFI	TLI
Reference value	<3	<0.1	<0.10	>0.9	>0.9	>0.9	>0.9	>0.9
Testing value	3.469	0.04	0.064	0.841	0.817	0.941	0.941	0.936

### 4.3 Hypothesis testing

The test results showed in Table 4.

**Table 4:** Test results of path relationship.

Assumptions	Path	Non-standardized coefficient	SE	*Z* (CR value)	*p*	Standardized coefficients	Hypothesis testing
H1	PC → JI	0.164	0.043	3.786	0.000	0.159	Set up
H2	PC → POS	0.706	0.031	23.029	0.000	0.686	Set up
H3	PC → PR	0.449	0.033	13.574	0.000	0.486	Set up
H4	PC → SE	0.316	0.028	11.424	0.000	0.424	Set up
H5	POS → JI	0.165	0.037	4.442	0.000	0.165	Set up
H6	PR → JI	0.513	0.034	14.915	0.000	0.460	Set up
H7	SE → JI	0.312	0.041	7.601	0.000	0.226	Set up

When professional commitment affected perceived organizational support, the standardized path coefficient value was 0.686 > 0, and this path showed a significant level of 0.01 (*Z* = 23.029, *p* = 0.000 < 0.01), indicating that professional commitment had a significant positive effect on perceived organizational support.

When professional commitment affected psychological resilience, the standardized path coefficient value was 0.486 > 0, and this path showed a significance at the 0.01 level (*Z* = 13.574, *p* = 0.000 < 0.01), indicating that professional commitment had a significant positive effect on psychological resilience.

When professional commitment affected self-efficacy, the standardized path coefficient value was 0.424 > 0, and this path showed a significant level of 0.01 (*Z* = 11.424, *p* = 0.000 < 0.01), indicating that professional commitment had a significant positive effect on self-efficacy.

When professional commitment affected job involvement, the standardized path coefficient value was 0.165 > 0, and this path showed a significance at the 0.01 level (*Z* = 4.442, *p* = 0.000 < 0.01), indicating that professional commitment had a significant positive effect on job involvement.

When perceived organizational support affected job involvement, the standardized path coefficient value was 0.159 > 0, and this path showed a significance at the 0.01 level (*Z* = 3.786, *p* = 0.000 < 0.01), indicating that perceived organizational support had a significant positive effect on job involvement.

When psychological resilience affected job involvement, the standardized path coefficient value was 0.460 > 0, and this path showed a significance at the 0.01 level (*Z* = 14.915, *p* = 0.000 < 0.01), indicating that psychological resilience had a significant positive effect on job involvement.

When self-efficacy affected job involvement, the standardized path coefficient value was 0.226 > 0, and this path showed a significance at the 0.01 level (*Z* = 7.601, *p* = 0.000 < 0.01), indicating that self-efficacy had a significant positive effect on job involvement.

### 4.4 Mediating effect test

This study uses the SPSS PROCESS macro developed by Hayes (2012) to test the mediation effects of professional commitment (PC) on job involvement (JI) through self-efficacy (SE), psychological resilience (PR), and perceived organizational support (POS). The results, as shown in Table 5, revealed the following: firstly, professional commitment had a direct significant positive effect on job involvement (*β* = 0.164, *p* < 0.000), confirming the main effect. Secondly, the mediating effects of self-efficacy, psychological resilience, and perceived organizational support were tested. Self-efficacy mediated 16.224% of the total effect, psychological resilience mediated 37.807%, and perceived organizational support mediated 19.117%, all showing partial mediation. Finally, to verify the robustness of these mediating effects, the Bootstrap method was used with 1,000 resamplings (Diciccio and Romano, 1988; Hayes and Rockwood, 2017). The results confirmed significant mediating effects within the 95% confidence intervals:

**Table 5:** Results of mediating effect test.

Path	Total effect	Mediate effect	S.D.	*z*-value	*p*-value	95% boot CI	Direct effect	Percentage	Result
PC → SE → JI	0.609**	0.099	0.020	4.874	0.000	0.053–0.134	0.164**	16.224%	Partial mediation
PC → PR → JI	0.609**	0.230	0.029	7.896	0.000	0.155–0.270	0.164**	37.807%	Partial mediation
PC → POS → JI	0.609**	0.117	0.030	3.916	0.000	0.048–0.170	0.164**	19.117%	Partial mediation

*indicates *p* < 0.05; **indicates *p* < 0.01; ***indicates *p* < 0.001.

Self-efficacy: indirect effect = 0.099, 95% CI [0.053, 0.134].

Psychological resilience: indirect effect = 0.230, 95% CI [0.155, 0.270].

Perceived organizational support: indirect effect = 0.117, 95% CI [0.048, 0.170].

This further validated the hypothesis that self-efficacy, psychological resilience, and perceived organizational support serve as significant mediators between professional commitment and job involvement.

## 5 Conclusion and discussion

### 5.1 Conclusion

Through the in-depth analysis of the psychodynamic relationships among professional commitment, self-efficacy, psychological resilience, perceived organizational support and job involvement of female teachers in Chinese primary schools, this study reveals the interaction and influence mechanism among these variables. The results show that professional commitment plays a central role in the job involvement of female elementary school teachers, and self-efficacy and psychological resilience, as individual psychological resources, play an important role in promoting professional commitment and job involvement.

On the one hand, there is a significant positive relationship between professional commitment and job involvement, indicating that the stronger the professional commitment, the higher the job involvement of teachers. This finding is consistent with the resource conservation theory that individuals tend to protect and accumulate resources that are valuable to them. In this study, professional commitment itself can be regarded as an important psychological resource, which affects teachers’ professional identity and satisfaction, and is also the core driving force of their daily work motivation.

On the other hand, perceived organizational support, as an external resource, can significantly enhance teachers’ professional commitment, and promote their job involvement by enhancing teachers’ self-efficacy and resilience. This suggests that educational administrators and policy makers should pay attention to establishing a supportive work environment to help teachers better cope with professional challenges and stimulate their inner potential by providing necessary material and emotional support.

In addition, psychological resilience and self-efficacy, as mediating variables, played an important role in the relationship between professional commitment and job involvement. This shows that in the process of improving the professional commitment and job involvement of female teachers in primary schools, we need to pay attention not only to their external environment and support, but also to the cultivation of their internal psychological resources.

**Figure 2:**
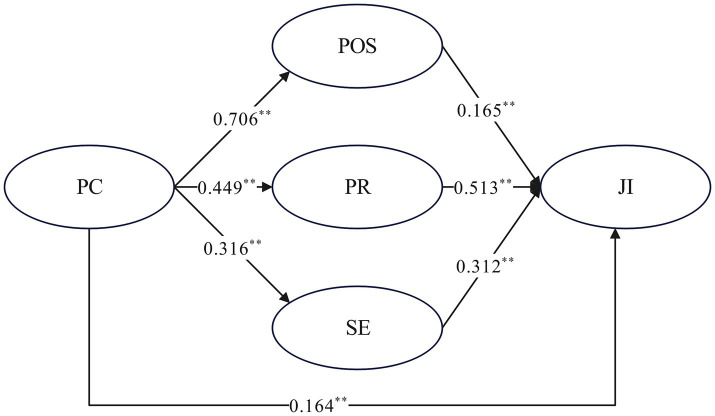
Psychodynamics of professional commitment and job involvement of female primary school teachers. *indicates *p* < 0.05; **indicates *p* < 0.01; ***indicates *p* < 0.001.

In conclusion, this study not only provides new insights for theory, but also provides important guidance for practical application, that is, through enhancing professional commitment, self-efficacy, psychological resilience and perceived organizational support, female teachers in primary schools can effectively enhance their job involvement, thus improving the quality of education and student development. This provides important implications for future educational practice and policy making (Figure 2).

### 5.2 Discussion

The findings of this study are consistent with and extend several prior studies on job involvement, professional commitment, and psychological resilience among teachers. For example, the positive relationship between self-efficacy and job involvement observed in this study aligns with the work of Tschannen-Moran and Hoy (2001), who found that teachers with higher levels of self-efficacy tend to be more engaged and committed to their work. Bakker and Demerouti (2008) also emphasized that teachers who believe in their ability to overcome challenges are more likely to experience higher levels of job satisfaction and involvement. Our findings build on these results by demonstrating that self-efficacy not only enhances job involvement but also mediates the relationship between professional commitment and perceived organizational support.

Furthermore, this study supports the conclusions of Wang (2024), who highlighted the importance of perceived organizational support in reducing teacher burnout and promoting well-being. Our results extend this by showing that organizational support plays a crucial role in enhancing resilience, which, in turn, positively influences job involvement. This reinforces the Conservation of Resources (COR) Theory proposed by Hobfoll et al. (2018), which posits that individuals who perceive strong organizational support are better able to mobilize internal resources like resilience to mitigate the negative effects of job stress.

However, our study diverges from previous research in certain areas. For instance, while Madigan and Kim (2021) focused on the role of resilience in reducing turnover intentions, our findings suggest that resilience plays a more direct role in enhancing job involvement. This highlights the importance of resilience not only as a protective factor against burnout but also as a proactive contributor to teacher engagement and performance.

This study addresses key gaps in the literature by exploring how self-efficacy, resilience, and organizational support interact to influence job involvement, particularly in the context of female primary school teachers in China. Previous research, such as that by Bakker and Demerouti (2008) and Skaalvik and Skaalvik (2014, 2017), has largely focused on Western educational settings, with limited attention to the unique socio-cultural challenges faced by teachers in non-Western contexts. By focusing on Chinese female teachers, this study contributes to a broader understanding of how cultural expectations and gender roles influence professional commitment and job involvement.

### 5.3 Inspiration

The results of this study provide important practical guidance for educational policy makers and school administrators to improve teachers’ job involvement and educational quality. The following are some implications based on the research results.

Strengthening teachers’ professional commitment: In view of the significant positive impact of professional commitment on job involvement, schools and educational institutions should strengthen teachers’ professional commitment through positive professional culture and recognition mechanism. For example, teachers’ recognition conferences can be held regularly to publicly praise teachers’ excellent teaching and innovative practices, so as to enhance teachers’ professional identity and satisfaction.

Provide comprehensive organizational support: Perceived organizational support is the key to enhance teachers’ professional commitment and job engagement. Administrators should ensure that teachers have access to necessary resources, including instructional materials, technical support, and professional development opportunities. At the same time, support for teachers’ personal lives, such as flexible working arrangements and mental health services, should be strengthened to help them better balance work and family life.

Develop teachers’ self-efficacy and psychological resilience: Educational institutions should set up special training programs aimed at improving teachers’ self-efficacy and psychological resilience. This can be achieved through workshops, seminars and team building activities, particularly those focused on teaching strategies, emotional management and stress coping skills.

Strengthen the communication between leaders and teachers: a good communication mechanism can enhance teachers’ organizational support. Leaders should regularly communicate with teachers to understand their needs and challenges and provide timely feedback and support. In addition, a transparent decision-making process and teacher participation mechanism should be established so that teachers feel that their voices are heard and valued.

Create a supportive work environment: Educational institutions should work to create an inclusive, supportive, and stimulating work environment. This includes implementing fair evaluation systems, providing career growth paths, and promoting diversity and inclusion in the workplace.

### 5.4 Future recommendations

While this study provides valuable insights into the relationship between professional commitment, perceived organizational support, self-efficacy, and job involvement among female primary school teachers in China, it also raises several important questions that future research can explore.

First, this study employed a cross-sectional design, which limits the ability to infer causality. Future research could benefit from employing a longitudinal approach to track changes in job involvement and professional commitment over time. Such studies could provide a more dynamic understanding of how self-efficacy and resilience develop as teachers gain more experience and face new challenges. Longitudinal studies could also explore how the cumulative effects of organizational support impact job involvement in the long term (Kline, 2015; Wang, 2024).

Second, while this research focused on female teachers in China, it would be valuable to extend this work to other cultural settings or different professional groups. For instance, future studies could investigate whether similar relationships exist in male teachers or in non-educational professions where job involvement and organizational support are also critical. By conducting cross-cultural comparisons, researchers can explore whether the findings of this study are generalizable across different educational systems or cultural contexts (Bakker and Demerouti, 2008).

Third, additional variables such as emotional intelligence, job satisfaction, or work-life balance could be integrated into future models to gain a more holistic understanding of the factors that influence teacher engagement. Emotional intelligence, in particular, has been found to play a crucial role in mitigating stress and enhancing resilience, and it would be valuable to see how this interacts with other psychological resources like self-efficacy (Tschannen-Moran and Hoy, 2001). Moreover, job satisfaction could serve as a potential mediator between professional commitment and job involvement, providing deeper insights into the motivational factors that drive teacher engagement (George et al., 2022).

Finally, this study opens the door to intervention-based research that explores strategies to enhance teachers’ resilience and self-efficacy through professional development programs or organizational support initiatives. Future researchers could design and implement interventions to test the effectiveness of resilience training or leadership development programs aimed at improving organizational support. Such interventions could offer practical solutions for educational institutions seeking to improve teacher well-being and performance (Hobfoll et al., 2018; Liu and Yu, 2024).

By exploring these areas, future studies can build on the findings of this research, contributing to a more comprehensive understanding of teacher job involvement and the factors that enhance professional commitment and well-being.

## Data Availability

The original contributions presented in the study are included in the article/supplementary material, further inquiries can be directed to the corresponding author.
